# New Insights into the Structure and Mode of Action of *Mo*-CBP_3_, an Antifungal Chitin-Binding Protein of *Moringa oleifera* Seeds

**DOI:** 10.1371/journal.pone.0111427

**Published:** 2014-10-27

**Authors:** Adelina B. Batista, José T. A. Oliveira, Juliana M. Gifoni, Mirella L. Pereira, Marina G. G. Almeida, Valdirene M. Gomes, Maura Da Cunha, Suzanna F. F. Ribeiro, Germana B. Dias, Leila M. Beltramini, José Luiz S. Lopes, Thalles B. Grangeiro, Ilka M. Vasconcelos

**Affiliations:** 1 Department of Biochemistry and Molecular Biology, Federal University of Ceará, Fortaleza, Ceará, Brazil; 2 Bioscience and Biotecnology Center, State University of North Fluminense, Campos dos Goytacazes, Rio de Janeiro, Brazil; 3 Physics Institute of São Carlos, University of São Paulo, São Carlos, São Paulo, Brazil; 4 Department of Biology, Federal University of Ceará, Fortaleza, Ceará, Brazil; Saint Louis University, United States of America

## Abstract

*Mo-*CBP_3_ is a chitin-binding protein purified from *Moringa oleifera* Lam. seeds that displays inhibitory activity against phytopathogenic fungi. This study investigated the structural properties and the antifungal mode of action of this protein. To this end, circular dichroism spectroscopy, antifungal assays, measurements of the production of reactive oxygen species and microscopic analyses were utilized. *Mo-*CBP_3_ is composed of 30.3% α-helices, 16.3% β-sheets, 22.3% turns and 30.4% unordered forms. The *Mo-*CBP_3_ structure is highly stable and retains its antifungal activity regardless of temperature and pH. *Fusarium solani* was used as a model organism for studying the mechanisms by which this protein acts as an antifungal agent. *Mo*-CBP_3_ significantly inhibited spore germination and mycelial growth at 0.05 mg.mL^−1^. *Mo*-CBP_3_ has both fungistatic and fungicidal effects, depending on the concentration used. Binding of *Mo*-CBP_3_ to the fungal cell surface is achieved, at least in part, via electrostatic interactions, as salt was able to reduce its inhibitory effect. *Mo*-CBP_3_ induced the production of ROS and caused disorganization of both the cytoplasm and the plasma membrane in *F. solani* cells. Based on its high stability and specific toxicity, with broad-spectrum efficacy against important phytopathogenic fungi at low inhibitory concentrations but not to human cells, *Mo*-CBP_3_ has great potential for the development of new antifungal drugs or transgenic crops with enhanced resistance to phytopathogens.

## Introduction

Plants use several strategies to overcome fungal attacks, including the production of antimicrobial peptides and proteins [1], [2]. Much effort has been dedicated to researching these bioactive constituents, particularly because the chemically-synthesized antifungal compounds used to prevent and contain these pathogens comprise a potential environmental threat [3], [4], [5]. In general, these defense-related proteins interfere with the fungal life cycle by either impairing growth or killing the pathogen [6], [7]. The antifungal properties of these proteins may be exploited for use in the development of transgenic crops that have enhanced resistance to phytopathogenic fungi [8].

Chitin-binding proteins (CBPs) represent a group of proteins also found in plants that often have a basic pI, a molecular mass ranging from 3.1 kDa up to 20 kDa, and high resistance to both extreme pH changes and proteolysis. Some CBPs have the ability to inhibit fungal growth [9], as they bind to and disrupt the proper function of chitin, a key component of the fungal cell wall [10]. It has been suggested that the binding of these proteins to chitin in filamentous fungi leads to the disruption of both cell wall biogenesis and cell polarity [11], [12].

Recently, our research group isolated a chitin-binding protein named *Mo*-CBP_3_ from *Moringa oleifera* Lam. seeds [13]. This protein is a basic glycoprotein (18 kDa by SDS-PAGE) and does not display haemagglutination, chitinase or β-1,3-glucanase activity. *Mo*-CBP_3_ presented potent antifungal activity against the phytopathogenic fungi *Fusarium solani*, *F. oxysporum*, *Colletotrichum musae* and *C. gloeosporioides* at a low concentration (0.05 mg.mL^−1^). The phytopathogenic effect of *Mo*-CBP_3_ against fungi was observed even when the protein was heated at 100°C for 1 h or pre-treated with 150 mM *N*-acetyl-D-glucosamine.

As *Mo*-CBP_3_ has a low molecular mass and is a protein with potent antifungal activity at low concentrations, it is a very promising bioactive candidate that may be explored to determine whether it can confer resistance against phytopathogenic fungi to economically and nutritionally important crops. To further test *Mo*-CBP_3_, it is essential to obtain additional knowledge about its structure and mode of action. Here, we report new structural features of *Mo*-CBP_3_ that reveal a correlation between its structural stability and its antifungal activity. In addition, to know about the mechanisms by which this protein acts as an antifungal agent, its ability to induce the endogenous production of reactive oxygen species and to trigger morphologic and ultrastructural alterations were analyzed using *F. solani* as a model. *F. solani* is an easy-to-handle and fast-developing species, making it ideal for *in* *vitro* assays, and it holds relevance as a phytopathogenic fungus that attacks economically important crop plants. Furthermore, to have a preliminary clue whether *Mo*-CBP_3_, as a chemical agent against fungi, displays cytotoxicity, the level of lysis of the human red blood cells was examined.

## Materials and Methods

### Biological materials and reagents

*M. oleifera* seeds were obtained from trees at the Campus do Pici of the Federal University of Ceará (UFC), Fortaleza, Brazil. A voucher specimen (No. EAC34591) was deposited in the Prisco Bezerra Herbarium, UFC. The filamentous fungus *F. solani* (URM 3708) was provided by the Departamento de Micologia of the Universidade Rural de Pernambuco, Recife, Brazil. All chemicals used were of analytical grade.

### *Mo*-CBP_3_ preparation

A highly purified *Mo*-CBP_3_ preparation devoid of contaminating proteins was obtained according to Gifoni et al. [13]. Mature seeds were ground in a coffee grinder, and the resulting flour was treated with *n*-hexane. Defatted flour was extracted with 50 mM Tris-HCl, pH 8.0, containing 150 mM NaCl (1∶10 w/v), for 4 h at 4°C under constant stirring and was then filtered through cheesecloth. After centrifugation at 15,000 *g* at 4°C for 30 min, the supernatant was exhaustively dialyzed against Milli-Q grade water and centrifuged again under the same conditions. (NH_4_)_2_SO_4_ was added to the soluble material, denoted as the albumins, to yield 90% saturation. This protein fraction (F0–90%) was then dissolved in and dialyzed against the extracting buffer and applied to a chitin column that had been equilibrated with the same buffer. After elution with the starting buffer of the unbound proteins from the column, the chitin-bound proteins, named P_NAG_ and P_AC_, were eluted with 100 mM *N*-acetyl-D-glucosamine (NAG) that was prepared in the extracting buffer and with 50 mM acetic acid (pH 5.0), respectively. The P_NAG_ sample was dialyzed against 100 mM acetic acid and distilled water, lyophilized and applied to a cation*-*exchange *matrix (*Resource S) that had been previously equilibrated with 50 mM sodium acetate buffer, pH 5.2. Four major adsorbed protein peaks (*Mo*-CBP_2_, *Mo*-CBP_3_, *Mo*-CBP_4_, and *Mo*-CBP_5_) were recovered after being selectively desorbed by stepwise elution with 400, 500, 600, and 700 mM NaCl, respectively, included in the equilibrium buffer. As *Mo*-CBP_3_ was purified to homogeneity, had high yield and presented the highest activity against the phytopathogenic fungi *Fusarium solani*, *Fusarium oxysporum*, *Colletotrichum musae* and *Colletotrichum gloesporioides*, as previously reported by our research group [13], it was used for further analyses. The purity of *Mo*-CBP_3_ was checked by denaturing gel electrophoresis [14]. The identity of *Mo*-CBP_3_ was confirmed by N-terminal amino acid sequence analysis by Edman degradation (Shimadzu PPSQ-10A automated protein sequencer).

### Protein concentration

The protein concentration was determined according to Bradford [15], using BSA as a standard. Absorbance at 280 nm was also used to monitor the protein elution profiles during chromatography.

### Far-UV circular dichroism (CD) spectroscopy

CD spectra measurements were made on a JASCO J-715 spectropolarimeter (Jasco Instruments, Tokyo, Japan) in an N_2_ atmosphere at 25°C. *Mo*-CBP_3_ (40 µg) was dissolved in 20 mM phosphate-buffered saline (PBS) at pH 7.0 and transferred to a rectangular quartz cuvette with a 0.1 cm path length. Eight scans were performed with a scan rate of 20 nm.min^−1^ and a 4 s response time. CD spectra were measured from 190 to 250 nm. The contributions of the secondary structural elements of *Mo*-CBP_3_ were determined by CD spectra deconvolution analyses using the basis reference protein set SMP56 of the CDPro software [16] and applying three methods, CONTIN/LL, SELCON 3 and CDSSTR. CD spectroscopy was also used to assess *Mo*-CBP_3_ thermal stability. To do this, *Mo*-CBP_3_ (40 µg in PBS) was heated gradually in 10°C increments from 26 to 90°C in a TC-100 circulating water bath (Jasco). The samples were maintained at each temperature for 10 min, and spectra were recorded from 190 to 250 nm. To evaluate structural stability as a function of pH, *Mo*-CBP_3_ (40 µg) was incubated for 240 min in 20 mM sodium acetate/phosphate/borate buffer at different pH values (2.0, 4.0, 6.0, 8.0, 10.0, and 12.0) before recording the CD spectrum.

### Effect of pH and temperature on the inhibition of the conidial germination of *F. solani* by *Mo*-CBP_3_

The filamentous fungus *F. solani* was grown in Petri dishes containing potato dextrose agar (PDA) medium for 12 days at room temperature (22°C). Fresh conidia suspensions were prepared by rinsing the surface of the 12-day-old sporulated cultures with sterile distilled water and the aid of a triangular Drigalski rod. Spore suspensions were filtered through cheesecloth in a laminar flux chamber under sterile conditions, and conidia were quantified using a Neubauer chamber under an optical microscope (Olympus System Microscope BX 60). Antifungal assays were conducted as described by Ji and Kuc [17]. For analyzing the changes in conidial germination as a function of pH, *Mo*-CBP_3_ samples, at antifungal concentration (0.1 mg.mL^−1^), were dissolved in 20 mM sodium acetate/phosphate/borate buffer at different pH values (2.0, 4.0, 6.0, 8.0, 10.0 and 12.0) and incubated with 10 µL of the conidia suspension (2×10^5^.mL^−1^) in reticulated plates. For the non-inhibitory controls, conidia were incubated in each buffer in the absence of *Mo*-CBP_3_. The plates were placed in a plastic box maintained near 100% relative humidity at 22°C in the dark for 24 h. After this time, 50 conidia were randomly selected from each treatment and evaluated for germination under an optical microscope. A conidium that had emitted a hyphae at least twice the length of the ungerminated conidium was considered to have successfully germinated. In parallel, to assess whether the ability of *Mo*-CBP_3_ to inhibit spore germination was affected by heat treatment, *Mo*-CBP_3_ was heated in a water bath at 100°C for 60 min, cooled on ice for 10 min, and assayed as described above. Each experiment was performed in triplicate, and images were taken with a digital camera (Sony, MCV-CD350 model, 14.2 megapixels).

### Effect of *Mo*-CBP_3_ on the mycelial growth and conidial viability of *F. solani*

A quantitative assay for fungal growth inhibition was performed following the protocol developed by Broekaert et al. [18]. A conidia suspension (2×10^5^ cells.mL^−1^) was incubated in 96-well flat microplates with 100 µL of potato dextrose broth in the absence of *Mo*-CBP_3_ and allowed to germinate for up to 12 h in the dark at 37°C. Next, 100 µL of different concentrations of *Mo*-CBP_3_ (0.05, 0.1, 0.5 and 1 mg.mL^−1^) were added. Cell growth was also determined without the addition of *Mo*-CBP_3_. Fungal growth was monitored by turbidimetry at 630 nm from 0 to 49 h using an automated microplate reader (Model Elx800, Bio-Tek Instruments). The absorbance values taken immediately after *Mo*-CBP_3_ addition were recorded and established as zero and were discounted from every readings taken onwards. To evaluate the conidial viability of *F. solani* after treatment with different concentrations of *Mo*-CBP_3_, 150 µL aliquots were taken from the wells, transferred to Eppendorf tubes and centrifuged at 3,000 *g* for 1 min at 25°C and the supernatant discarded. The remaining conidia were washed with sterile distilled water to remove *Mo*-CBP_3_, reculturing in Petri dishes containing PDA medium and kept in an incubator at 27°C. Images of the mycelium growth were taken after 5 days. All experiments were carried out in triplicate.

### Evaluation of the electrostatic interaction of *Mo*-CBP_3_ with the conidial membrane

To evaluate the presence of electrostatic interactions between *Mo*-CBP_3_ and the conidial plasma membrane, *Mo*-CBP_3_ (0.1 mg.mL^−1^) was first dissolved in solutions with different NaCl concentrations (25, 75 and 150 mM) and antifungal assays were performed following the methodology described in Section 2.5. In the negative, non-inhibitory controls, conidia were incubated only in 25, 75 and 150 mM NaCl, all in the absence of *Mo*-CBP_3_. As a positive inhibitory control, *Mo*-CBP_3_ was used at antifungal concentration (0.1 mg.mL^−1^). All experiments were carried out in triplicate.

### Evaluation of reactive oxygen species (ROS) production by *F. solani* conidia after *Mo*-CBP_3_ treatment

To evaluate the ability of *Mo-*CBP_3_ to induce the endogenous production of ROS in *F. solani* conidia, the *in* *situ* assay described by Thordal-Christensen et al. [19], with some modifications [20], was conducted, using 3,3′-diaminobenzidine (DAB). *F. solani* conidia (2×10^6^ cells.mL^−1^) were incubated with *Mo-*CBP_3_ (0.1 mg.mL^−1^) prepared in H_2_O, with only H_2_O or bovine serum albumin (BSA, 0.1 mg.mL^−1^ in H_2_O) used as controls, all in the presence of DAB (0.5 mg.mL^−1^ in H_2_O). After 1 h incubation, aliquots of conidial suspensions were placed on glass slides and examined under a light microscope (Olympus System Microscope BX 60).

### Scanning electron microscopy (SEM)

To analyze the *F. solani* conidial morphology after treatment with *Mo-*CBP_3_, the fungal cells (2×10^6^ conidia.mL^−1^) were incubated in either the absence or presence of *Mo-*CBP_3_ (0.05 mg.mL^−1^ in H_2_O). After 48 h incubation, the cells were harvested and fixed for 30 min at 25°C with 2.5% (v/v) fresh glutaraldehyde and 4% (v/v) paraformaldehyde prepared in 50 mM cacodylate buffer, pH 7.2. Subsequently, the materials were rinsed three times with the above buffer, post-fixed for 30 min at 25°C with 1% (m/v) osmium tetroxide (OsO_4_) solution diluted in the same buffer and rinsed with distilled water. After that, conidia were dehydrated in a graded acetone series (30, 50, 70, 90, and 100%; v/v), critical-point dried in CO_2_, coated with 20 nm gold and observed in a Zeiss 962 scanning electron microscope.

### Transmission electron microscopy (TEM)

Structural changes of *F. solani* conidium induced by *Mo-*CBP_3_ were assessed by TEM. Conidia were grown for 48 h in water in either the presence (0.05 mg.mL^−1^) or absence of *Mo-*CBP_3_ and processed as for SEM analysis. After post-fixation in 1% (m/v) OsO_4_ and dehydration in a graded acetone series, the specimens were embedded in Epon resin (Polybeded 812). Ultrathin sections (0.1 µm) were fixed onto copper grids, stained with uranyl acetate (10 min) and lead with citrate (5 min). Visualization of cells was performed in a transmission electron microscope (Zeiss TEM 900) operating at 80 kV.

### Haemolytic assay

This was carried out using human red blood cells (hRBCs) collected from healthy donors in heparinized tubes [21]. hRBCs were separated from plasma by centrifugation (3,000 *g*, 10 min, 25°C) and washed three times with 100 mM sodium phosphate buffer, pH 7.4, containing 150 mM NaCl (PBS). A 1% (v/v) suspension was prepared and incubated for 1 h at 37°C with serial dilutions of *Mo-*CBP_3_ (from 280 to 0.137 µM) in PBS. After the incubation period, suspensions were centrifuged at 3,000 *g* for 10 min at 25°C, aliquots of the supernatants were transferred to Eppendorf tubes and the absorbances taken at 405 nm (spectrophotometer Novaspec II, Pharmacia) to monitor the release of haemoglobin. Triton X-100 and PBS were used as positive (100% haemolysis) and negative controls, respectively. The haemolysis percentage was calculated using the following equation: Haemolysis (%) = [A*_protein_*–A*_PBS_*]/[A*_Triton_*–A*_PBS_*], where A means absorbance at 405 nm.

## Results and Discussion

### *Mo*-CBP_3_ preparation

**Figure 1:**
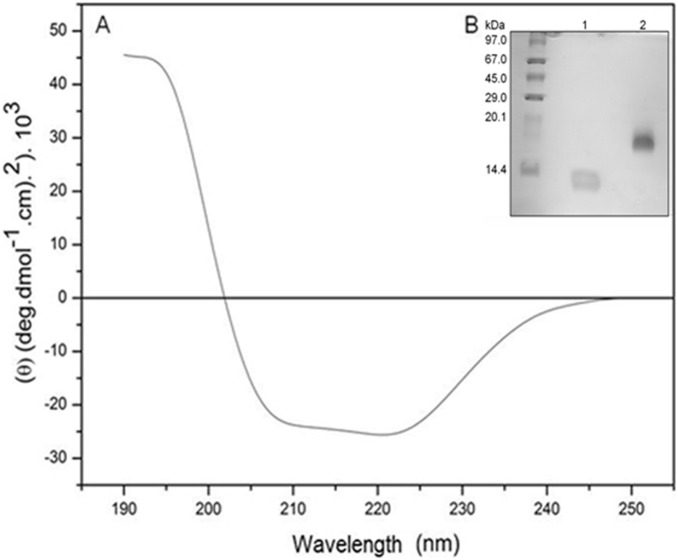
Structural properties of *Mo*-CBP_3_. (A) Circular dichroism spectra (Far-UV) of *Mo*-CBP_3_ (2.22 mM) in 20 mM sodium phosphate buffer, pH 7.0, using a rectangular quartz cuvette with a 0.1 cm path length. (B) Denaturing polyacrilamide gel electrophoresis (SDS-PAGE - 15% acrylamide gel) of *Mo-*CBP_3_. Molecular mass standards are shown (in kDa) on the left; Lanes 1 and 2, *Mo-*CBP_3_ (20 µg) in reducing (4 kDa and 8 kDa subunits) and non-reducing conditions (18 kDa), respectively.

The *Mo*-CBP_3_ preparations used in the present study were confirmed to be homogeneous and free of contaminants, as shown in a representative Figure 1. *Mo*-CBP_3_ is an 18 kDa protein that inhibits the conidia germination of *F. solani*, *F. oxysporum, C. musae* and *C. gloeosporioides*, confirming the findings of Gifoni et al. [13]. The purity of *Mo*-CBP_3_ was further proved by N-terminal sequencing analysis.

### Structure-antifungal activity relationships

The far-UV CD spectra of native *Mo*-CBP_3_ showed minima at approximately 208 and 222 nm (Figure 1). The deconvolution of the CD spectra performed using the CDPro package [16] revealed the following content of the secondary structure fraction: 30.3% α-helices, 16.3% β-sheets, 22.3% turns and 30.4% unordered forms. Therefore, *Mo*-CBP_3_ can be classified as an alpha-beta protein [22].

**Figure 2:**
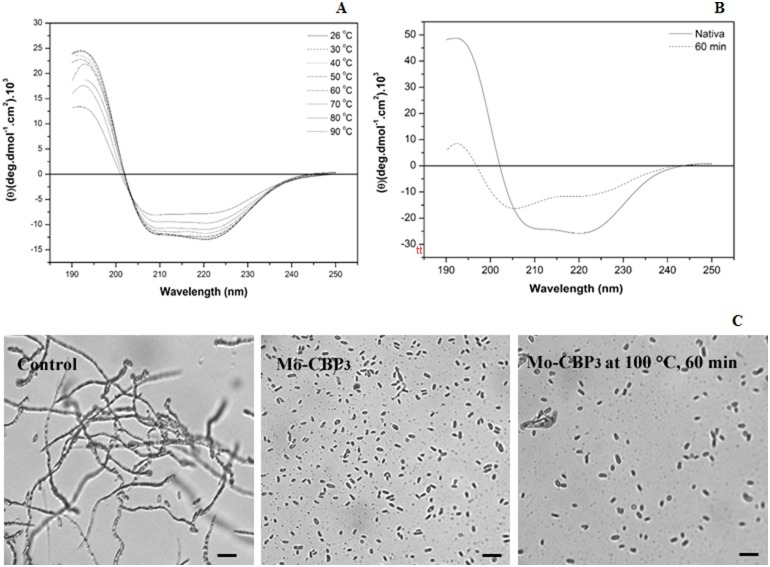
Effect of temperature on the conformation and antifungal activity of *Mo*-CBP_3_. (A) Far-UV CD spectra of *Mo*-CBP_3_ (2.22 mM) at various temperatures. (B) CD spectra of *Mo*-CBP_3_ (2.22 mM) after heating at 100°C for 60 min. (C) Light micrographs of *F. solani* spores in either the culture medium (control) or incubated with *Mo*-CBP_3_ (0.1 mg.mL^−1^) and either unheated or previously heated at 100°C for 60 min in a water bath. Trials were conducted for 24 h at 22°C in the dark. Bars: 2.5 µm.

**Figure 3:**
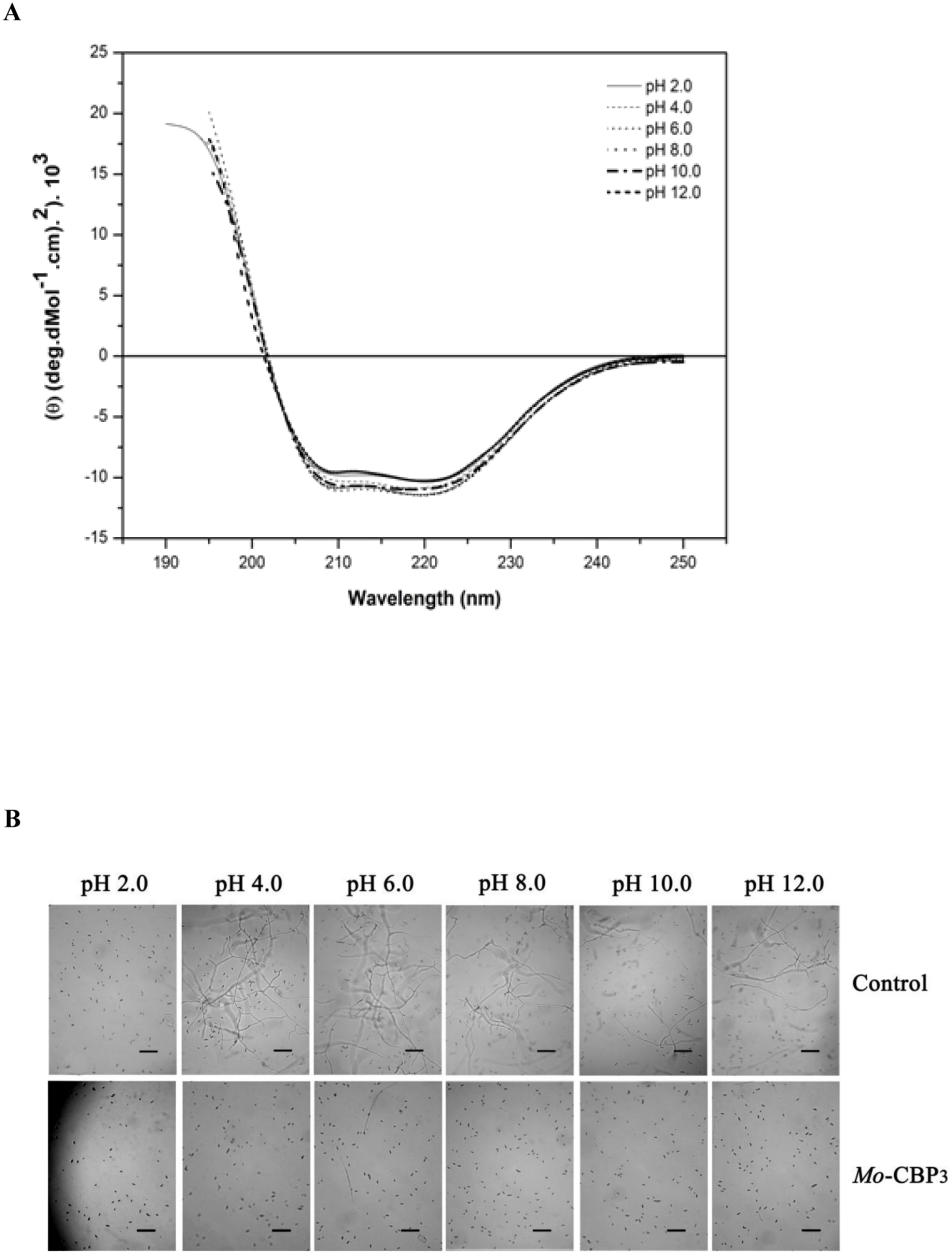
Effect of pH on the conformation and antifungal activity of *Mo*-CBP_3_. (A) Far-UV CD spectra of *Mo*-CBP_3_ (2.22 mM) at various pH values. (B) Light micrographs of *F. solani* spores either in 20 mM sodium acetate-borate-phosphate buffer at different pH values (control) or incubated with *Mo*-CBP_3_ (0.1 mg.mL^−1^) prepared in these buffers. Trials were conducted for 24 h at 22°C in the dark. Bars: 2.5 µm.

To further characterize *Mo*-CBP_3_, the effects of temperature and pH on its secondary structure and antifungal activity were evaluated. No significant changes in the CD spectra of *Mo*-CBP_3_ were observed after heat treatment at 90°C for 10 min (Figure 2A). After heat treatment at 100°C for 60 min, the CD spectra of *Mo*-CBP_3_ demonstrated only a discrete alteration (Figure 2B). Similarly, *Mo*-CBP_3_ was still able to inhibit the spore germination of *F. solani* after heating at 100°C for 60 min. Additionally, the CD spectral shape did not change from pH 2.0 to pH 12.0 (Figure 3A), suggesting that the protein structure is maintained and that even the pH extremes were insufficient to alter the net charge of *Mo*-CBP_3_ in a way to cause electrostatic repulsion, with later rupture of the hydrogen bonds. To correlate the structure of *Mo*-CBP_3_ to its antifungal activity, *Mo*-CBP_3_ was dissolved in 20 mM sodium acetate-borate-phosphate buffer at different pH values, and the antifungal activity on *F. solani* spore germination was tested. The inhibitory activity of *Mo*-CBP_3_ was similar at all pH ranges tested (4.0, 6.0, 8.0, 10 and 12.0) (Figure 3B). However, it was not possible to evaluate the behavior of the protein at pH 2.0, as spore germination did not occur even in the control, most likely because this pH is very acidic and has an adverse effect on the development of *F. solani*. These data demonstrate that *Mo*-CBP_3_ exhibits high resistance to both temperature and pH changes, thus retaining its antifungal activity. The elevated structural and functional stability of *Mo*-CBP_3_ can be attributed to the presence of cysteine residues in its structure. *Mo*-CBP_3_ is a chitin-binding protein, and many proteins for which the amino acid sequences are known that possess this property share a common structural domain composed of 43 amino acids, with many cysteine and glycine residues in conserved positions [23]. In fact, of the 22 identified residues from the N-terminus of *Mo*-CBP_3,_ 27.3% were cysteines [13]. The presence of such residues can lead to formation of disulphide bridges, making these proteins more resistant to denaturation [9], [24]. In fact, the reduction of these disulphide bridges abolished the antifungal activity of *Mo*-CBP_3_. Several disulphide bonds are present in osmotins and thaumatin-like proteins, and it is thought that they contribute to the high structural stability of these proteins [25].

### Effect of *Mo*-CBP_3_ on the mycelial growth and conidial viability of *F. solani*

**Figure 4:**
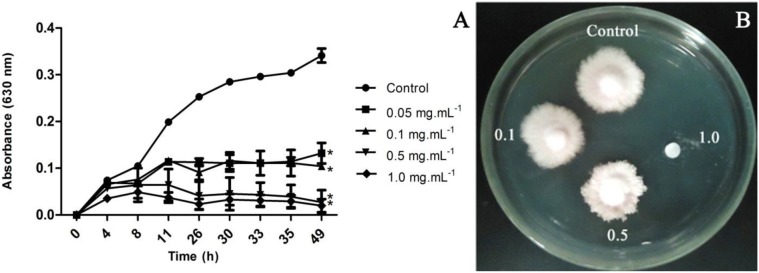
Effect of *Mo*-CBP_3_ on the mycelial growth and conidial viability of *F. solani*. (A) Mycelial growth of *F. solani* in the presence of *Mo*-CBP_3_. A conidium suspension (2×10^5^ cells.mL^−1^) was incubated in the absence of *Mo*-CBP_3_ and allowed to germinate for up to 12 h in the dark at 37°C. Next, 100 µL of different concentrations of *Mo*-CBP_3_ were added. The fungal culture in the absence of *Mo*-CBP_3_ was used as control. Each point is the mean of three estimates. The values are means (± SD) of triplicates. Asterisks indicate significant differences (*P*<0.05) compared to control group (Tukey’s Test). (B) Effects of *Mo*-CBP_3_ (0.1, 0.5 and 1.0 mg.mL^−1^) on the conidium viability of *F. solani* after inhibition growth assay.

In addition to inhibiting spore germination, the ability of *Mo*-CBP_3_ to affect fungal growth was analyzed. *Mo*-CBP_3_ was inhibitory to the mycelial mass development of *F. solani* in comparison to the control incubated in the absence of *Mo*-CBP_3_ (Figure 4A). *Mo*-CBP_3_ displayed a significant inhibitory effect on fungus growth at a concentration of only 0.05 mg.mL^−1^, with close to 62% inhibition within 49 h. At higher concentrations (0.5 and 1.0 mg.mL^−1^), the antifungal effect was observed at earlier stages. For example, at 1.0 mg.mL^−1^, *Mo*-CBP_3_ inhibited 94% of the growth of *F. solani* 26 h post-incubation. In reality, *Mo*-CBP_3_ behaves both as fungistatic and fungicidal protein, depending on its concentration and the stage of fungus development. Pre-incubation of *F. solani* spores with *Mo*-CBP_3_ at concentrations up to 0.5 mg.mL^−1^ for 49 h followed by removal of the protein restored the mycelial growth capacity of the fungus, indicating the fungistatic effect of *Mo*-CBP_3_ at low concentrations. In contrast, prior incubation of *F. solani* spores with 1.0 mg.mL^−1^ *Mo*-CBP_3_ followed by removal of the protein abolished (100% inhibition) the fungus viability, as mycelial growth was inhibited (Figure 4B), which characterizes *Mo*-CBP_3_ fungicidal action. It is well known that several chitin-binding proteins have antifungal activity [26]. A chitin-binding lectin from *Setcreasea purpurea* (SPL) causes inhibition of *Rhizoctonia solani*, *Penicillium italicum*, *Sclerotinia sclerotiorum*, and *Helminthosporium maydis* at 1.51 mg.mL^−1^ [27]. However, it is remarkable that the inhibitory effects of *Mo*-CBP_3_ on *F. solani* growth were more pronounced in this study than those observed by Gifoni et al. [13]. The differences observed are presumably due to the different protocols used. For example, the growth inhibition assays in the present study were performed in liquid medium, differing from the previous study, which was made on solid medium (Petri dish). In addition, it is worth noting that the cultivation medium has a great influence on the antifungal activity of *Mo*-CBP_3_, as *F. solani* growth inhibition could be detected only when yeast extract was not present in the medium composition. Based on these data, it is plausible to speculate that in the presence of yeast extract, which contains cell wall fragments and negatively charged mannan [28], the interaction of *Mo*-CBP_3_ with the filamentous fungus would be compromised, affecting its antifungal activity.

### Mode of action of *Mo*-CBP_3_ upon fungal cell

**Figure 5:**
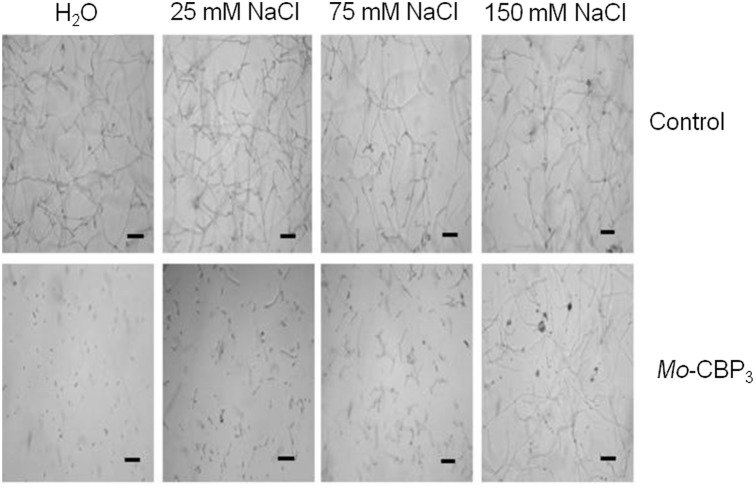
Effect of NaCl on the antifungal activity of *Mo*-CBP_3_. Light micrographs of *F. solani* spores in either H_2_O or different NaCl concentrations (control), with or without incubation with *Mo*-CBP_3_ (0.1 mg.mL^−1^) prepared in these solutions. Trials were conducted for 24 h at 22°C in the dark. Bars: 2.5 µm.

Antimicrobial molecules possess several features to fulfill their role in plant defense mechanisms. For rapid killing, antimicrobial molecules often act at the cell surface rather than the cell interior [29]. Therefore, it was hypothesized that besides the binding of *Mo*-CBP_3_ to the fungus chitin, since it is a chitin-binding protein as established by affinity chromatography [13], *Mo*-CBP_3_ could also bind to *F. solani* cell membrane components, at least in part, via electrostatic interactions. This prediction is supported by the observation that NaCl at 25, 75 and 150 mM reduced the inhibitory effect of *Mo*-CBP_3_ (0.1 mg.mL^−1^) on *F. solani* spore germination in comparison to the controls in the absence of *Mo*-CBP_3_ and presence of the same above concentrations of NaCl (Figure 5). Sensitivity to ionic strength with loss of antimicrobial activity has been described for other basic antimicrobial proteins and peptides that are thought to act via electrostatic interactions with negatively charged membrane components, as well as for those that bind to specific receptors [30], [31]. In the *F. solani* membrane, such electrostatic interactions of *Mo*-CBP_3_, which is a cationic protein, most likely occur with the negatively charged phospholipid phosphatidylinositol [32].

**Figure 6:**
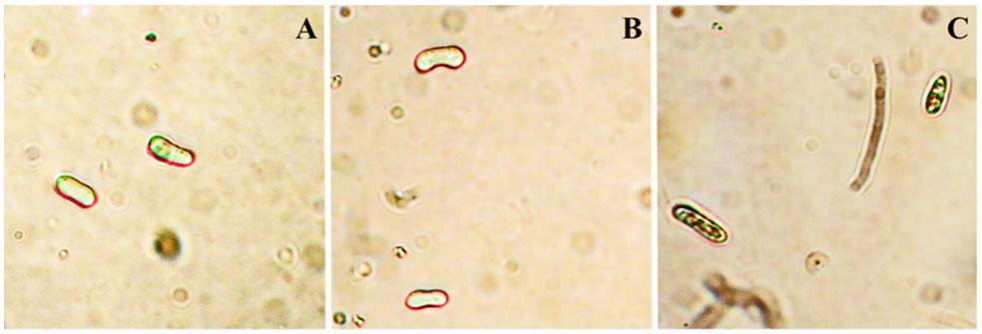
Induction of reactive oxygen species (ROS) in *F. solani* spores. Cells were treated with 3,3′-diaminobenzidine (DAB) for ROS detection. Cells were previously incubated with (A) H_2_O, (B) BSA (0.1 mg.mL^−1^) or (C) *Mo*-CBP_3_ (0.1 mg.mL^−1^). Uptake of DAB is confirmed by the dark staining (reddish-brown) reaction in conidia, as indicated by arrows. Bars: 2.5 µm (A–C).

After this initial binding to components of the fungal membrane, secondary effects that are induced internally in the cell were investigated. Figure 6C shows that *Mo*-CBP_3_ (0.1 mg.mL^−1^) induced ROS production as reveled by the presence of a reddish-brown pellet inside the *F. solani* spores, in contrast to the negative controls, H_2_O and BSA (0.1 mg.mL^−1^) (Figures 6A and 6B, respectively). The ROS induction capacity of various antifungal peptides and proteins has been previously reported. Similar to *Mo*-CBP_3_, the defensin from *Phaseolus vulgaris* (*Pv*D_1_) causes ROS induction in *F. solani* cells at 0.1 mg.mL^−1^ [33]. Another example is the *Raphanus sativus* antifungal peptide 2 (*Rs*-AFP_2_), which is able to stimulate ROS production in *Candida albicans* in a dose-dependent manner, but is unable to do so in an *Rs*-AFP_2_-resistant Δ*gcs C. albicans* mutant that lacks the *Rs*-AFP_2_-binding site in its membranes [34]. This finding suggests that upstream binding of the macromolecule is needed for ROS production. An increase in the generation of ROS that exceeds the cellular neutralization capacity of the fungus promotes oxidative stress and may cause the hyperoxidation of proteins, lipids and nucleic acids and consequently cell death [35].

**Figure 7:**
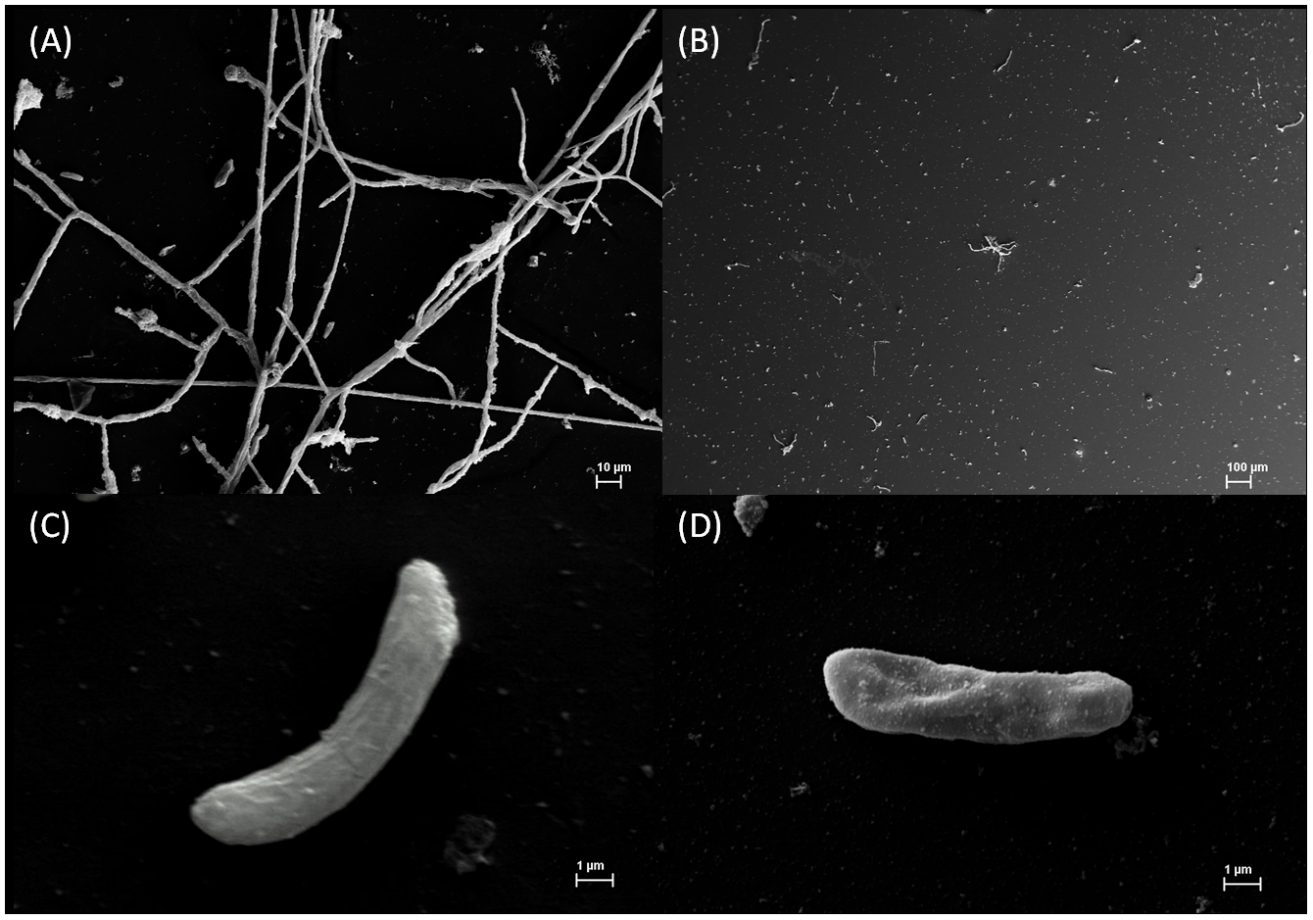
Scanning electron microscopy of *F. solani* cells. The cells were cultured either in the absence (A, C) and presence (B, D) of *Mo*-CBP_3_ (0.05 mg.mL^−1^). In (A) the fungus cell has typical growth and developed hyphae in contrast with (B) which shows ungerminated spores and spores that emitted the germination peg, but not developed further. The zooming image of a *Mo*-CBP_3_ treated spore (D) shows typical alterations in the cell surface morphology in contrast with control spore (C).

SEM was employed to allow visualization of any morphological changes promoted by *Mo*-CBP_3_ on *F. solani* cells. Photomicrographs of *F. solani* conidia were taken 48 h after growth in the presence or absence of *Mo*-CBP_3_ (0.05 mg.mL^−1^). Normal hyphal growth was observed in the control cells (Figure 7A), but not in in the cells treated with *Mo*-CBP_3_ (Figure 7B). Closer examination of *F. solani* cells treated with *Mo*-CBP_3_ revealed loss of asymmetry, deformations and wrinkles in comparison to control cells, as represented in Figures 7D and 7C, respectively. Similar alterations were detected in *S. cerevisiae* cells after incubation with a 2S albumin-homologous protein (0.1 mg.mL^−1^) from passion fruit seeds [36].

**Figure 8:**
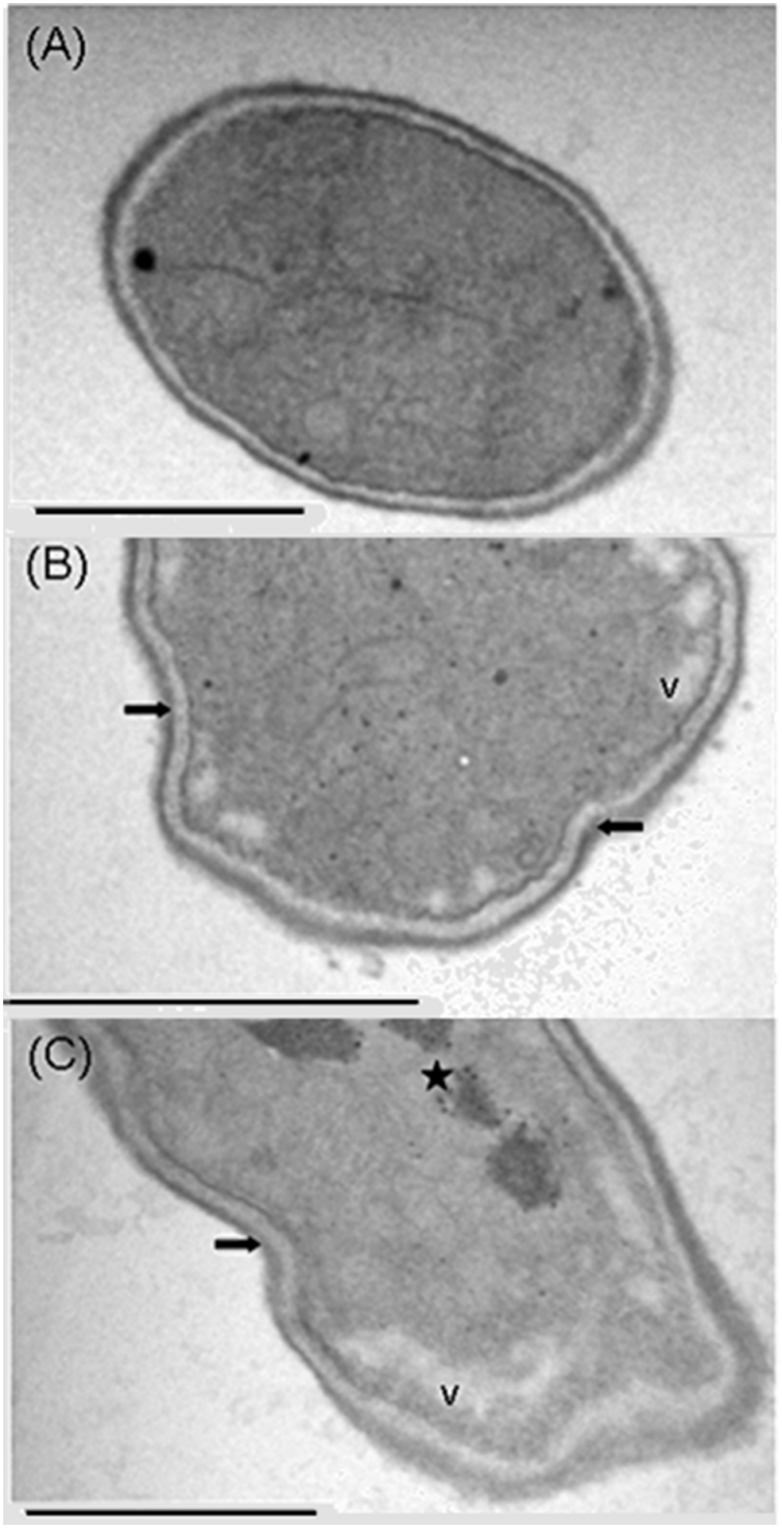
Transmission electron microscopy of *F. solani* cells. The cells were cultured either in the absence (A) and presence (B and C) of *Mo*-CBP_3_ (0.05 mg.mL^−1^). Star indicates condensation of the cytosolic content. Vacuole condensation (V) is also shown. Arrows indicate shrinkage of the cell wall. Bars: 0.5 µm (A–C).

Ultrastructural analysis of *F. solani* cells also revealed alterations in the presence of Mo-CBP_3_ (0.05 mg.mL^−1^). It was observed condensation of the cytosol content, vacuolation and shrinkage of the cell wall (Figures 8B and C) when compared with control cells (Figure 8A). Vacuoles serve as compartments either for storage of resources or for detoxification purposes [37]. Thus, possibly the increased vacuole formation in the fungus cell might be related to a defense response of *F. solani* to the toxic effects of Mo-CBP_3_. In addition to these above changes observed, notable accumulation of electrodense granular material was observed in the cytosol of the cells incubated with Mo-CBP_3_ (Figure 8C). It is plausible to speculate that the electrodense granular material observed might result from the electrostatic interactions of the cationic Mo-CBP_3_ with negatively charged primary or secondary metabolites present into the fungus cell, based on the coagulant (flocculent) properties of this protein as previously reported [13]. These alterations in *F. solani* morphology as visualized by TEM are typically found in cells that have undergone apoptosis [38]. This is in agreement with the results shown above, as ROS are classical apoptotic markers [39]. Thus, these data together suggest that the antifungal properties of *Mo*-CBP_3_ are triggered by alterations in the cell surface. Brul et al. [40] found that the filamentous fungi *Penicillium roqueforti*, *Trichoderma harzianum*, *Paecilomyces variotii*, *Aspergillus niger*, and *A. nidulans* allow molecules up to 150 kDa to cross the cell wall. Thus, it cannot be ruled out that *Mo*-CBP_3_ may eventually pass through the cell wall barrier, interact with the cell membrane receptors and induce secondary effects internally in *F. solani* to promote cell death.

### Evaluation of cytotoxicity effects of *Mo*-CBP_3_

**Figure 9:**
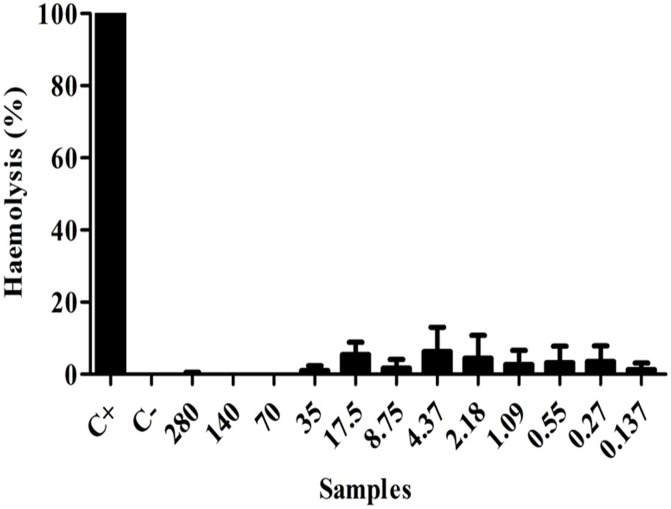
Evaluation of the cytotoxic effect of *Mo*-CBP_3_. *In vitro* haemolytic activity of *Mo*-CBP_3_ on human erythrocytes using concentrations ranging from 280 to 0.137 µM. Positive control (C+): 1% Triton X-100. Negative control (C–): 100 mM sodium phosphate buffer, pH 7.4, 150 mM NaCl.

Many antimicrobial proteins also exhibit toxic potential on eukaryotic cells. In this context, the mechanical stability of the membrane of red blood cells is a good indicator to evaluate *in* *vitro* the effects of various compounds when screening for cytotoxicity [41]. These cells may undergo a loss of membrane integrity and die rapidly as a result of cell lysis. Thus, to evaluate whether *Mo*-CBP_3_ causes cytotoxicity, haemolytic assay was utilized by measuring the release of haemoglobin at different *Mo*-CBP_3_ concentrations. *Mo*-CBP_3_ was compared with the detergent Triton X-100, whose relative haemoglobin release was set at 100%. For all concentrations tested (from 0.137 a 280 µM) *Mo*-CBP_3_ did not show haemolytic activity (Figure 9), suggesting that the antifungal action of this protein occurs via a selective interaction with the fungal membrane. This result shows that although *Mo*-CBP_3_ displayed remarkable antifungal activity against phytopathogenic fungi, it shows no haemolytic activity. Similar results were found for an antifungal peptide (AFP-J) purified from potato tubers (*Solanum tuberosum* cv. L Jopung) [42].

In conclusion, this study reinforces previous data on the antifungal properties of *Mo*-CBP_3_ and reports new information about its structural features and mode of action. The CD spectral data from different temperature and pH conditions indicate that the high structural stability of *Mo*-CBP_3_ results in the effectiveness of its antifungal activity through interactions with the cell membrane, which causes prominent morphological changes followed by the induction of oxidative stress, eventually leading to cell death. Considering its elevated stability and specific toxicity, with broad-spectrum efficacy against important phytopathogenic fungi at low inhibitory concentrations but not to human cells, *Mo*-CBP_3_ has great potential for the development of new antifungal drugs or transgenic crops with enhanced resistance to phytopathogens.
